# Evaluating [^68^Ga]-Ga PSMA PET/CT for Detecting Prostate Cancer Recurrence Post-High-Intensity Focused Ultrasound and Brachytherapy: A Single-Center Retrospective Study

**DOI:** 10.3390/curroncol32010009

**Published:** 2024-12-26

**Authors:** Andrea Di Giorgio, Marco Rapa, Simona Civollani, Andrea Farolfi, Stefano Fanti

**Affiliations:** 1 Nuclear Medicine, Alma Mater Studiorum, University of Bologna, Via Massarenti 9, 40138 Bologna, Italy; marco.rapa@studio.unibo.it (M.R.); stefano.fanti@aosp.bo.it (S.F.); 2 Department of Medical Physics, IRCCS, Azienda Ospedaliero-Universitaria di Bologna, 40138 Bologna, Italy; simona.civollani@aosp.bo.it; 3 Nuclear Medicine, IRCCS, Azienda Ospedaliero-Universitaria Di Bologna, 40138 Bologna, Italy; andrea.farolfi@aosp.bo.it

**Keywords:** prostate cancer, PET, PSMA, HIFU, brachytherapy, nuclear medicine, oncology

## Abstract

Focal therapy offers a promising approach for treating localized prostate cancer (PC) with minimal invasiveness and potential cost benefits. High-intensity focused ultrasound (HIFU) and brachytherapy (BT) are among these options but lack long-term efficacy data. Patient follow-ups typically use biopsies and multiparametric MRI (mpMRI), which often miss recurrences. PET/CT with PSMA has emerged as a promising tool for detecting residual disease or recurrence post-treatment, offering higher sensitivity and specificity than traditional imaging. We retrospectively reviewed patients who underwent [^68^Ga]Ga-PSMA-11 PET/CT for biochemical recurrence (BCR) after HIFU or brachytherapy from 2016 to 2024. Out of 22 patients, 32% had HIFU and 68% had brachytherapy. The median time from treatment to PET scan was 77 months, with a median PSA level of 3 ng/mL. [^68^Ga]Ga-PSMA-11 PET/CT identified PC recurrence in 63.6% of cases. Of these, 50% showed prostate recurrence, 14% had lymph node involvement, and 28% had metastatic disease. Focal therapies like HIFU and brachytherapy are effective and minimally invasive options for localized PC. [^68^Ga]Ga-PSMA-11 PET/CT is valuable for detecting recurrence or residual disease, enhancing post-treatment surveillance.

## 1. Introduction

Among the therapeutic options for localized prostate cancer (PC), focal therapies represent a promising approach due to their limited treatment-related side effects [1]. In contrast, radical prostatectomy (RP) and radiation therapy (RT), which are standard treatments for organ-confined disease, may lead to side effects such as incontinence and erectile dysfunction [2].

Brachytherapy (BT) and—even more so—high-intensity focused ultrasound (HIFU) are non-invasive therapies that have been shown in many studies to have minimal impact on patients’ quality of life, significantly reducing the occurrence of adverse effects, with a 7-year disease-free survival rate of 69% [3]. An additional advantage of HIFU is the possibility of repeating the treatment in case of therapeutic failure or disease recurrence. Currently, there is no reliable method for managing follow-up: prostate-specific antigen (PSA) monitoring, based on the Phoenix [4] and Stuttgart criteria [5], has low specificity and sensitivity; multiparametric MRI (mpMRI) is limited by tissue changes induced by therapies [6]; repeated prostate biopsies, the most accurate option, remain invasive and poorly tolerated by patients, especially in the long term [7].

PET/CT with prostate-specific membrane antigen (PSMA) is an undeniable tool to detect recurrence in PC and it has demonstrated to have strong potential in detecting recurrence or residual disease after brachytherapy and HIFU treatments [8]. It offers high sensitivity and specificity for identifying prostate cancer lesions, including those missed by traditional imaging methods. To date, no studies have evaluated the role of [^68^Ga]Ga-PSMA-11 PET/CT in identifying the site of relapse in patients undergoing focal therapy for prostate cancer. However, some promising smaller studies have explored the use of [^68^Ga]Ga-PSMA-11 PET/MRI in this setting [9,10]. In this study, we examined cases of disease recurrence following HIFU and BT that underwent PET-PSMA at our center to assess its role in post-treatment surveillance and its effectiveness in the early detection of recurrence or residual disease.

## 2. Materials and Methods

We retrospectively analyzed patients with a history of PC who underwent [^68^Ga]Ga-PSMA-11 PET/CT for biochemical recurrence (BCR) after HIFU or BT, according to the EAU and EAMN guidelines [6,11]. Patients were screened at our institution from 2016 to 2024 and clinical data were extracted from electronic medical records. PET images were reviewed by three expert nuclear medicine physicians who applied the PROMISE criteria [11].

### 2.1. PET/CT Images Acquisition

[^68^Ga]Ga-PSMA-11 (Glu-NH-CO-NH-Lys-(Ahx[^68^Ga(HBED-CC)]) was used as the PSMA tracer. The median injected activity was 162 MBq (IQR range, 150–168 MBq). The median tracer uptake period was 67 min (IQR, 64, 25–75, 25 min). We acquired images using a PET/CT scanners; specifically, the Discovery MI (GE Healthcare, Boston, MA, USA), Discovery STE (GE Healthcare, Boston, MA, USA), and uVista (United Imaging Healtcare, Shangai, China) were used. A full-dose CT scan (15–400 mA, 120 kV) without contrast media was performed. The PET image acquisition included a whole-body scan (pelvis to vertex, 2–3 min/bed position depending on the patient weight). All PET images were reconstructed using attenuation, dead-time, random-event, and scatter corrections. PET images were reconstructed with an iterative algorithm (ordered-subset expectation maximization) in an axial 256 × 256 matrix on the Discovery MI (GE) (8 subsets, 4 iterations, 88FWHM), 128 × 128 matrix on the Discovery STE (GE) (20 subsets, 2 iterations, 6FWHM) and 128 × 128 matrix on the uVista (United) (20 subsets, 2 iterations, 3 FWHM).

### 2.2. Image Analysis

Three independent blinded readers performed the whole-body PET image analysis on a per-region basis (T, N, M1a, M1b, M1c) following the PROMISE v2 criteria [11]. Since there is no strict guideline for the positivity rate after focal therapy, interpretation was done only based on readers’ experience. A centralized majority rule was applied to determine the final positivity rate.

### 2.3. Data Analysis

Collected data were exported in an Excel (Microsoft, Redmond, WA, USA) spreadsheet, and were analyzed. Descriptive analysis was performed using SPSS software 30.0.0 (IBM, Armonk, NY, USA).

## 3. Results

**Figure 1:**
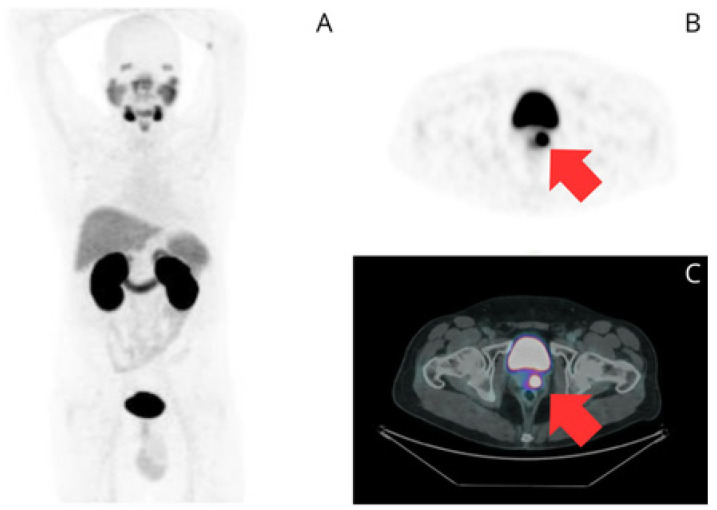
A 56-year-old male with Gleason 3 + 3 PC. His initial PSA was 8.4 ng/mL. He underwent HIFU in February 2022 which involved a left intermediate-basal quadrantectomy and right peripheral zonal ablation. His PSA lever after HIFU was 2.65 ng/mL. Twelve months later, he experienced BCR with a PSA rise to 4.35 ng/mL. A [^68^Ga]Ga-PSMA-11 PET/CT scan was performed in April 2023 to evaluate for recurrence. The maximum intensity projection (**A**), transaxial PET image (**B**), and fused transaxial PET/CT image (**C**) show a focal area of intense uptake in the left lobe (red arrows) with a maximum standardized uptake value (SUVmax) of 22, confirming local recurrence.

In our retrospective study, we evaluated patients who underwent [^68^Ga]Ga-PSMA-11 PET/CT at our institution between 2016 and 2024. We identified 22 patients with a history of primary localized prostate cancer (PC) who had undergone HIFU or brachytherapy (Table 1). These patients did not have significant oncological comorbidities. The median PSA value at diagnosis was 8 ng/mL (95% CI 4.2–10.5). The population included 7 (32%) patients with low-risk PC (ISUP Grade Group 1), 2 (9%) with intermediate risk (ISUP Grade Group 2), 6 (27%) with intermediate unfavorable risk (ISUP Grade Group 3), 3 (14%) with high risk or very high risk (ISUP Grade Group 4 or 5) and for 5 (23%) patients ISUP Grade Group was not known. A total of 7 patients (32%) underwent HIFU, while 15/22 (68%) underwent brachytherapy. Among these patients, 1 underwent HIFU after radical prostatectomy (RP) and 4 received hormonal therapy after focal therapy. The median time elapsed from the initial procedure to the PET scan was 77 months (95% CI 17–90) and no treatments were performed between the primary treatment and the PET/CT scan (Table 2). The median PSA value at the time of investigation was 2.8 ng/mL (95% CI 1–4). The [^68^Ga]Ga-PSMA-11 PET/CT was able to identify the site of PC recurrence in 63.6% (14/22) of patients with BCR (Table 2). Of the 14 patients with positive PSMA PET/CT scans, 7/14 (50%) were found to have local recurrence at the prostate (Figure 1) with a median SUVmax of 13 (95% CI 6–16). According to PROMISE v2 criteria, of the patients with positive PSMA PET/CT, 3 (21%) patients had a PSMA expression score (PSMAes) of 3, 4 (29%) patients with PSMAes 2, and 7 (50%) patients with PSMAes 1 [11]. Two patients (2/14, 14%) had a recurrence in the pelvic lymph nodes with median SUVmax of 4 (95% CI 3–9) while 4/14 (28%) had distant metastatic localization with a median SUVmax of 8 (95% CI 7–9). Following the PSMA PET/CT, 4/22 (18%) patients underwent re-biopsy. Histopathological analysis revealed ISUP Grade Group 3 in two patients and ISUP Grade Group 4 in the two remaining patients. The median follow-up from the date of the PET/CT scan was 55 months (95% CI 49–64.5). The median PSA value at the last follow-up was 0.1 ng/mL (95% CI 0.01–0.54).

## 4. Discussion

Technological improvements have led to the development of numerous non-invasive, targeted, and focal treatment modalities for PC [1,3,5]. Among these, HIFU has emerged as an innovative therapy for low-risk, localized PC. It has demonstrated both long-term efficacy and a favorable safety profile. Additionally, HIFU can be repeated without compromising its effectiveness in controlling the disease [12].

Similarly, BT represents another highly effective focal treatment option for PC, particularly in the management of recurrent disease [13]. BT has shown comparable 5-year recurrence-free survival (RFS) rates to other treatments, such as external beam radiation therapy (EBRT) (52%), RP (54%), HIFU (53%), and cryotherapy (50%) [13]. Moreover, BT has been associated with a lower incidence of severe genitourinary (GU) toxicity compared to these other modalities, with rates reported at 12% for BT, 21% for RP, 23% for HIFU, and 15% for cryotherapy [13].

The follow-up of patients who have undergone focal therapies remains a challenging issue. Current consensus guidelines recommend regular prostate biopsies, the monitoring of PSA levels, and the use of mpMRI [6]. However, each of these modalities has significant limitations. Prostate biopsies are poorly tolerated by patients and may lead to adverse events such as bleeding and GU infections. PSA levels are often unreliable in this context due to the presence of residual prostate tissue, which physiologically produces PSA. The Phoenix criteria, which define biochemical failure after focal therapies as a PSA value exceeding the nadir plus 2 ng/mL [4], and the later Stuttgart criteria, which adjust this threshold to the PSA nadir plus 1.5 ng/mL for patients treated with focal therapy [5], have not sufficiently resolved the issue, resulting in suboptimal sensitivity (65%) and specificity (77%). Additionally, mpMRI is often hindered by tissue alterations caused by focal treatments, particularly local hemorrhages.

In 2018, Burger et al. evaluated the role of [^18^F]F-Choline PET/MR in detecting local recurrence after HIFU therapy in PC; this single report was the first indicating that 18F-choline PET/MRI successfully localized recurrence after HIFU, showing a strong correlation with template biopsy results [9]. This hybrid system can enhance specificity, as MRI helps differentiate recurrence from benign prostate hyperplasia.

[^68^Ga]Ga-PSMA PET radiotracer has emerged as a key diagnostic tool in PC, across all stages, from staging to recurrence, with its high sensitivity [8]. Some authors have started to evaluate its role in biochemical recurrence after focal therapies as well. While [^68^Ga]Ga-PSMA PET/CT demonstrates high sensitivity and specificity in detecting prostate cancer lesions, it is essential to acknowledge potential pitfalls. The physiological uptake of PSMA in organs such as the salivary glands, kidneys, small bowel, and liver can potentially obscure or mimic malignant lesions [14,15]. Careful interpretation by experienced nuclear medicine physicians, in conjunction with other clinical and imaging findings, is crucial to minimize the risk of false negatives or false positives.

In 2019, Burger et al. were the first to report on [^68^Ga]Ga-PSMA-11 PET/MR in this setting, studying 10 patients who presented with biochemical recurrence after HIFU, with positive template biopsy but negative mpMRI [10]. Their initial findings suggest that [^68^Ga]Ga-PSMA-11 PET/MR may effectively detect prostate cancer recurrence after HIFU, which was not visible on mpMRI.

Later, in 2023, Duan et al. performed [^68^Ga]Ga-PSMA-11 and [^68^Ga]Ga-RM2 PET/MRI to identify the dominant lesion for HIFU and then to verify the response to treatment [16]. They showed that baseline and post-treatment SUV parameters, along with the primary score, can predict the biochemical response and treatment failure 12 months after treatment completion.

Conversely, very few studies have investigated the role of PET in detecting PC recurrence after brachytherapy for localized disease. The largest study to date, conducted by Mena et al., utilizing [^18^F]DCFPyL PET/CT, demonstrated a remarkably high detection rate of 94.6% [17].

**Figure 2:**
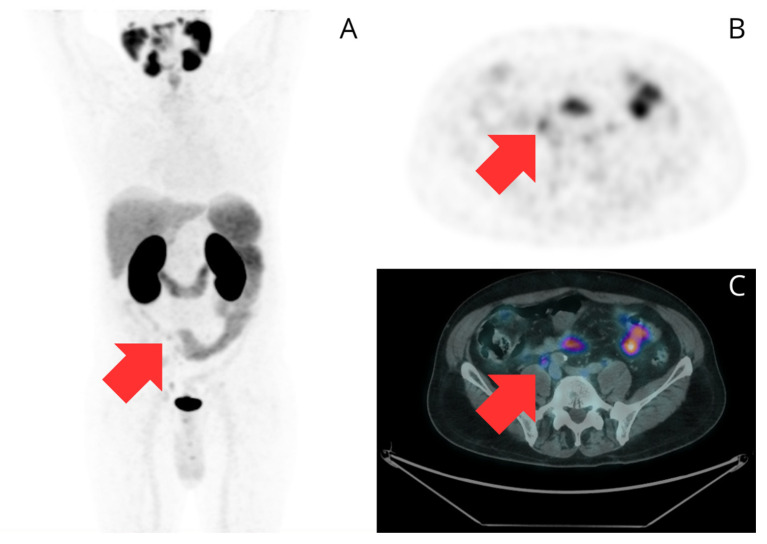
A 75-year-old male with a history of Gleason 3 + 4, ISUP 2 PC, and a baseline PSA of 9.3 ng/mL. He was treated with brachytherapy as his primary treatment. At 92 months after treatment, he experienced BCR with a PSA level of 1.74 ng/mL. A [^68^Ga]Ga-PSMA-11 PET/CT scan was performed to evaluate for recurrence. Maximum intensity projection (**A**), transaxial PET image (**B**), and fused transaxial PET/CT image (**C**) show an area of focal uptake in the right common iliac region (red arrows) corresponding to a millimetric lymph node, which is suggestive of lymph node recurrence (N1).

**Figure 3:**
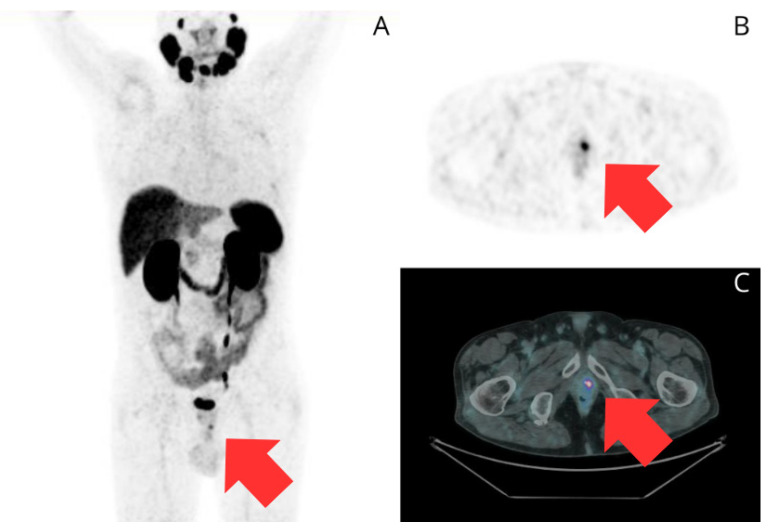
A 68-year-old male with a history of Gleason 3 + 3 PC and a baseline PSA of 7.4 ng/mL. He was treated with brachytherapy as his primary treatment. His post-treatment PSA nadir was 1.1 ng/mL. Subsequent follow-up involved PSA level monitoring. At 72 months after treatment, he experienced BCR with a PSA level of 3.19 ng/mL. A [^68^Ga]Ga-PSMA-11 PET/CT scan was performed to evaluate for recurrence. Maximum intensity projection (**A**), transaxial PET image (**B**), and fused transaxial PET/CT image (**C**) show two focal areas of intense uptake: one in the right lobe (SUVmax = 6.3) and another at the apex (red arrows) (SUVmax = 12), suggestive of local recurrence.

In our retrospective analysis, we found that [^68^Ga]Ga-PSMA-11 PET/CT was able to identify the site of recurrence in 14 out of 22 patients (64%). More specifically, the scans were positive in 6 out of 7 (86%) patients who underwent HIFU (Figure 2), and in 8 out of 15 (56%) who underwent BT (Figure 3). Considering the median pre-imaging PSA level of 2.8 ng/mL and the Stuttgart definition of BCR, this result is very promising. Additionally, the most common site of relapse was in the prostate (57%), which is comparable to other studies using different radiotracers and/or imaging tools [9,10,16,17], suggesting that [^68^Ga]Ga-PSMA-11 PET/CT is also reliable. We also noted that the scans identified nodal (N) lesions in 28% of cases and distant metastases (M) in 36%. Because of its high sensitivity in detecting secondary lesions related to PSA elevation, PSMA PET/CT can serve as a comprehensive modality capable of identifying both local and distant recurrence, with significantly lower costs compared to PET/MRI.

This study has several limitations. First, the number of patients is small, which restricts any statistical consideration, and it represents a highly heterogeneous population, as is often the case in retrospective studies. Second, many of the patients with positive PET/CT findings did not undergo magnetic resonance imaging or biopsy to confirm disease recurrence. This is partly due to the inherent challenges associated with obtaining repeat biopsies in this patient population. Prostate biopsies are invasive procedures that can cause discomfort, bleeding, and infection, and may be poorly tolerated by patients, particularly those who have already undergone focal therapy. Furthermore, the presence of post-treatment changes in the prostate gland can make it difficult to obtain accurate biopsy results. Finally, this study was conducted at a single institution, which may limit the generalizability of the findings to other settings.

Our data, consistent with both completed and ongoing prospective studies, are very promising in supporting [^68^Ga]Ga-PSMA PET/CT as a method for identifying disease recurrence after focal therapy in patients with localized prostate cancer. These findings should be confirmed and validated by prospective, multicenter studies with larger populations.

## 5. Conclusions

This study highlights the potential of [^68^Ga]Ga-PSMA-11 PET/CT as a valuable tool in the post-treatment surveillance of patients who have undergone focal therapies for localized prostate cancer. Our findings demonstrate its ability to detect sites of recurrence with reasonable accuracy, even in cases where conventional imaging modalities and PSA levels may be inconclusive. The ability of PSMA PET/CT to identify both local and distant recurrence further underscores its utility in guiding subsequent treatment decisions. While larger prospective studies are needed to validate these findings, our results suggest that [^68^Ga]Ga-PSMA-11 PET/CT could play a crucial role in improving the management and outcomes of patients with recurrent prostate cancer after focal therapy.

## Figures and Tables

**Table 1:** Summary of patient demographics, clinical characteristics, and treatment modalities.

Characteristic	Data	
*n*	22	
Age (*y*)	67 (63–71)	
PSA at PET/CT (ng/mL)	2.8 (1.5–3.7)	
Biopsy, ISUP grade group		
1	7	(32%)
2	2	(9%)
3	6	(27%)
4	2	(9%)
5	1	(5%)
Unknown	4	(18%)
Type of treatment		
HIFU	7	(32%)
Brachytherapy	15	(68%)
Continuous data are median and range		

**Table 2:** PET/CT scan results and TNM re-staging.

Characteristic	Data	
*n*	22	
Time from treatment to PET/CT scan (mo)	72 (17–90)	
PSA at PET/CT scan (ng/mL)	2.8 (1.5–3.7)	
PET/CT findings		
Positive	14	(64%)
Negative	8	(36%)
Positive scans after HIFU	6	(86%)
Positive scans after brachytherapy	8	(53%)
TNM (*n* = 14)		
*T*		
miT2	3	(21%)
miT3	5	(36%)
*N*		
miN1	3	(21%)
miN2	1	(7%)
*M*		
miM1a	4	(29%)
miM1b	1	(7%)
TNM classification based on PROMISE criteria [11]		
Continuous data are median and range		

## Data Availability

The raw data supporting the conclusions of this article will be made available by the authors upon request.
